# Ultraviolet radiation changes plant color

**DOI:** 10.1186/s12870-020-02471-8

**Published:** 2020-06-03

**Authors:** Kim Valenta, Kristin Dimac-Stohl, Frances Baines, Todd Smith, Greg Piotrowski, Norman Hill, Jonas Kuppler, Omer Nevo

**Affiliations:** 1 Department of Anthropology, University of Florida, Turlington Hall, PO Box 117305, Gainesville, FL 32611-7305 USA; 2 Department of Evolutionary Anthropology, Duke University, 130 Science Drive, Durham, NC 27708 USA; 3 UV Guide UK, Greenfield, School Lane, Govilon, Abergavenny NP79NT, Wales, UK; 4 Duke University Phytotron, 14 Circuit Dr, Durham, NC 27710 USA; 5 Ulm University, Institute of Evolutionary Ecology and Conservation Genomics, Albert-Einstein-Allee 11, 89081 Ulm, Germany

**Keywords:** Plant adaptive responses, Plant reflectance, Plant reproduction, Ultraviolet radiation, UV-B

## Abstract

**Background:**

Plant absorption of ultraviolet (UV) radiation can result in multiple deleterious effects to plant tissues. As a result, plants have evolved an array of strategies to protect themselves from UV radiation, particularly in the UV-B range (280–320 nm). A common plant response to UV exposure is investment in phenolic compounds that absorb damaging wavelengths of light. However, the inverse phenomenon – plant reflectance of UV to protect plant tissues – has not previously been explored. In a paired experiment, we expose half of our sample (*N* = 108) of insect-pollinated plants of the cultivar *Zinnia* Profusion Series to UV radiation, and protect the other half from all light < 400 nm for 42 days, and measure leaf and flower reflectance using spectroscopy. We compare UV-B reflectance in leaves and flowers at the beginning of the experiment or flowering, and after treatment.

**Results:**

We find that plants protected from UV exposure downregulate UV-B reflectance, and that plants exposed to increased levels of UV show trends of increased UV-B reflectance.

**Conclusions:**

Our results indicate that upregulation of UV-B reflecting pigments or structures may be a strategy to protect leaves against highly energetic UV-B radiation.

## Background

Exposure to the ultraviolet (UV) radiation in natural sunlight is unavoidable because of the need for plants to capture light for photosynthesis. Although it is recognized that exposure to UV radiation, particularly in the UV-B range of the spectrum (280–320 nm) plays an important regulatory role in plant development [1], UV-B radiation is also a significant plant stressor that causes deleterious effects [2]. Numerous studies have identified plant responses to UV-B radiation, in part prompted by the anthropogenic reduction in stratospheric, UV-filtering ozone [3]. Plant responses to UV-B exposure have been documented in multiple systems, and include tissue necrosis, and the activation of pathogen-defense and wound-signalling pathways [4, 5], regulatory changes, alterations in transpiration and photosynthesis, and changes in growth, development and morphology [6, 7], including plant reproductive processes [8, 9]. In addition, several studies have found that the negative effects of UV-B stress are increased when coupled with other stress factors such as herbivory, high temperatures, and drought [10, 11].

A major defense mechanism against UV-B radiation is the increased production of flavonoids, and other phenolic compounds in plant epidermal layers to provide UV-absorbing sunscreen [7, 10, 12–16]. This response is highly flexible and can occur within minutes to hours [7]. The inverse phenomenon may also occur; namely increased reflectance in UV-B as a means of reflecting and thereby protecting plant tissues from the deleterious effects of UV-B absorption, though a previous study of six plant cultivars did not detect it [16]. This hypothesis is supported by the finding that while fruit and leaf reflectance tend to be independent of one another between 400 and 700 nm, reflectance across much of the UV spectrum (300–400 nm) has been found to be positively correlated across multiple species and systems [17]. This finding indicates the potential importance of UV-reflectance across different plant parts as a strategy to protect plants against UV radiation. However, to our knowledge, the hypothesis that leaf UV reflectance serves as a protection against UV-B radiation has not been tested directly.

Reflectance of UV-B could generate downstream effects on ecological processes such as plant-insect herbivory, and pollination, but has received relatively little attention [8, 18]. Existing studies of the effects of UV radiation on ecological processes have documented decreased insect herbivory with increased plant UV-B exposure [19–22]. While these studies identify numerous biochemical mechanisms underlying ecological effects, the potential for changes in signals and cues that are visually salient to foraging insects and vertebrates remains unexplored [9]. This is despite the fact that numerous species of both vertebrate and invertebrate predators and pollinators readily distinguish UV-B reflectance [23–25], and demonstrate behavioral responses to it [23, 26].

Here, we address the hypothesis that plant reflectance in the UV-B part of the spectrum may be an adaptive response to UV-B exposure. We test the prediction that UV-B radiation drives synthesis of UV-B reflecting pigments in leaves, and thus in its absence there will be a decrease in leaf UV-B reflectance. We tested this in the hybrid cultivar *Zinnia* Profusion Fire, a fast-growing, insect-pollinated, flowering annual plant. We exposed 108 plants to two treatments: UV-exposed, and UV-deprived. Both groups were exposed to an identical light regime, but the UV-deprived plants had all ultraviolet radiation (< 400 nm) filtered out. Using spectroscopy, we monitored leaf and flower reflectance of UV-B radiation throughout a 42-day growth period. We discuss our findings in light of the potential role of UV in reflectance on plant predation and plant reproduction within the context of sensory ecology.

## Methods

We planted 108 seedlings of *Zinnia* Profusion Fire (Park Seed, S.C.) in 4P soil (Fafard), with one plant per 10 cm pot, with a depth of 8.9 cm, and fertilized each plant once using 1 Tbsp. Osmocote® time-release fertilizer granules (N-P-K ratio = 18–6-12). All seedlings were placed in a Model M-13 reach-in environmental growth chamber (Environmental Growth Chambers, Chagrin Falls, OH) at the Duke University Phytotron (1.2 m wide × 0.91 m deep × 1.0 m tall), and kept at 22 °C, with ambient humidity and CO_2_ levels. Ambient lighting was provided by a ceramic metal halide lamp (Philips MasterColor CDM-T Elite Med Wattage 315 watt lamp, Koninklijke Philips N.V, Amsterdam, 1096 BC Netherlands) providing full spectrum visible light. Light levels in the chamber were set to 250 μmol m-2 s-1 from 7:00–21:00 daily. All plants were divided into one of the two treatment categories, checked twice daily, and watered as needed. Half of the plants (*N* = 54) were placed under a PVC frame draped with a UV filter sheet (UV226, Epak Electronics Ltd., Chard, UK), which reduces UV radiation (< 400 nm) by 99%. The other half of plants were placed under an identical frame, draped with an otherwise identical sheet, but which permits UV radiation to pass (E130, Epak Electronics Ltd., Chard, UK).

Ultraviolet radiation in chambers was supplemented by suspending two 10% UV-B fluorescent tubes used for reptile care (Mystic Lamp, Big Apple Pet Supply, Inc., Boca Raton, FL) from the ceiling of the chamber, at a height of 56 cm above the chamber base. A spectral analysis of a lamp of the same brand was obtained using a USB2000 spectrometer with a UV-B compatible fibre-optic sensor with cosine adaptor, calibrated for absolute irradiance (Ocean Optics Inc., Dunedin, FL). Readings were also taken from this lamp using a total UV-B meter and a UV Index meter (Solarmeter 6.2 and 6.5 respectively, Solar Light Company, Glenside, PA 19038). This lamp emitted total UV-B levels similar to levels of direct sunlight (255 μW/cm^2^ at 15 cm, 142 μW/cm^2^ at 25 cm) however, the spectrum was very unlike sunlight, with a much greater proportion of the UV-B in the shorter wavelengths, and with non-terrestrial UV-B as low as 280 nm (Fig. S1), resulting in UV Index readings of UVI 30.4 at 15 cm and UVI 16.6 at 25 cm. Although the total UV-B readings would be classified as “moderate” by the EPA if they were from sunlight, with this spectrum the classification would be one of extreme exposure.

As the plants grew, the UV bulbs stayed in place at 56 cm from the chamber base, while the plant trays and the PVC frame supporting the films were moved to accommodate plant growth. The top of each plant remained within 15–25 cm of UV lights throughout the course of the study (film first height = 28 cm from chamber base, second film height = 40 cm from chamber base, chamber base to top of plants ~ 24 cm).

We measured the reflectance spectra of the two leaves growing at the top of each plant twice per week for 42 days, over 12 sampling sessions using a Jaz Portable Spectrometer with a PX-2 pulsed xenon light source (Ocean Optics Inc., Dunedin, FL), emitting a D-65 light source relative to a Spectralon white reflectance standard (Labsphere, North Sutton, NH). Reflectance measures were collected using UV-sensitive fiber optic probes fixed at a 45° angle, and external light was blocked using thick black felt. We measured the reflectance spectra of two flower petals per plant using the same sampling protocol, but because flowers were not present on all plants until 32 days into sampling, flower reflectance was only analyzed over the last 10 days of the sampling period, for a total of four sampling sessions. Flower petals were uniformly colored to the human eye, and reflectance measurements were taken from the centermost point of each petal. All sampling sessions were conducted between 8:00 am - 1:00 pm, and each day the order in which flowers were sampled was random. The proportion of UV-B in each sample was calculated as the area between 280 and 320 nm divided by the total area under the curve between 280 and 700 nm. For each individual, we used the average of the two measures from the same sampling day. All data are available in SI.

**Fig. 1:**
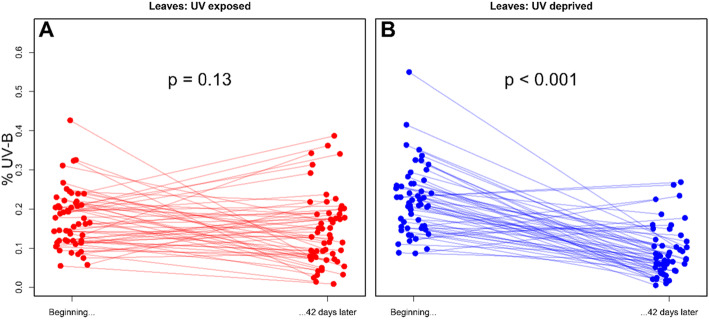
Percent UV-B Reflectance in Leaves. UV-B reflectance (270-320 nm) as a percentage of overall reflectance (280-700 nm) in leaves **a** exposed to increased UV radiation, and **b** protected from UV radiation over a 42 day period. *P* values refer to differences between reflectance at planting, and reflectance at the end of the experiment

To assess whether plants changed leaf or flower UV-B reflectance, we compared the first and last sampling days. This was done mainly because although randomly divided between the treatment groups, the groups differed in their initial UV-B reflectance (Fig. 1). We therefore opted for modelling the trend of change in each group rather than comparing the groups directly, since the different starting position could bias the results. We used a non-parametric Wilcoxon paired test to model the tendency of individuals to change their UV-B reflectance. Analysis was conducted in R 3.4.3 [27].

## Results

In the plants exposed to UV radiation, UV-B reflectance in leaves at the end of the experiment was not significantly different from UV-B reflectance at the time of planting (Wilcoxon paired test, two tailed: V = *N* = 54, 920, *p* = 0.13). In contrast, the plants that were protected from UV exposure significantly decreased the proportion reflectance of leaves in the UV-B range by the end of the sampling period (V = 1415, *N* = 54, *P* < 0.001; Fig. S1).

**Fig. 2:**
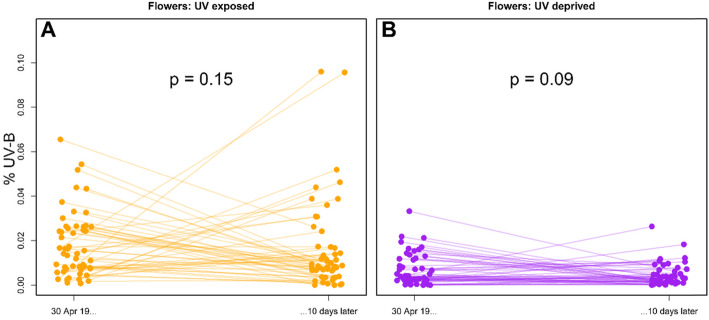
Percent UV-B Reflectance in Flowers. UV-B reflectance (270-320 nm) as a percentage of overall reflectance (280-700 nm) in flowers **a** exposed to increased UV radiation, and **b** protected from UV radiation over a 42 day period. Alpha values refer to differences between reflectance at day 32 of sampling – the first sample day on which all plants had flowers – and the last day of sampling 10 days later

In UV-exposed plants, there was a trend towards increased UV-B reflectance in flowers, but at the conclusion of the experiment, this increase was not significantly higher from the first day in which flowers were sampled (*N* = 52, V = 848, *P* = 0.15). Similarly, flowers protected from UV showed a trend of reduced UV-B reflectance, but the difference was not significant (*N* = 50, V = 812, *P* = 0.09; Fig. 2).

## Discussion

Our results demonstrate that in the absence of UV radiation, plants decrease proportional leaf reflectance in the UV-B part of the spectrum. This is in line with the hypothesis that plant adaptive response to increased UV radiation is increased reflectance, particularly in the UV-B part of the spectrum, where absorption can be particularly deleterious [2]. Our results also demonstrate plasticity in UV-B reflectance, as plants adjusted the reflectance of their leaves in response to light conditions. These results are in agreement with other studies that have documented plasticity in epidermal UV transmittance, even over short periods of time [7]. While our data does not allow us to accurately model the process of plant adaptation to their respective UV regime, they do indicate that the response is gradual. By chance, at the beginning of the study the UV-exposed group had leaves with lower UV-B reflectance than the UV-deprived group. Despite some fluctuations, reflectance remained on a similar level throughout the experiment. In contrast, UV-B reflectance of the leaves of the UV-deprived group started declining in the first few days of the experiment and ended lower than the UV-exposed group (Fig. 1).

We found similar trends, although not statistically significant, in flower reflectance (Fig. 2). It is possible that the effect is weaker because flowers were not present until relatively late in the experiment – the first flowers appeared 24 days into the 42 total sampling days. If indeed UV-B reflectance is downregulated in UV-protected flowers as well, it may imply that the reflectance there serves as a defense barrier similar to leaves. Alternatively, it is possible that the effect is pleiotropic, i.e., that UV reflectance in flowers may play a role in pollinator attraction, and its downregulation as a response to light environments is a costly side effect.

Our study did not aim at identifying the mechanism through which our model plant regulates UV-B reflectance. It has been suggested that both pigments (flavonoids, carotenoids) and physical structures such as leaf hairs or wax structures may be responsible for UV-B reflectance [28, 29], and our data do not allow us to determine which of these is responsible for the effects we observed. It is also important to note that our study used a UV-B source with a spectrum very unlike sunlight, exposing the plants to a much more extreme UV environment than they would receive under normal conditions. Despite this, the plants appeared to grow normally and leaf damage was not seen. However, future studies should utilise UV-B sources with spectra similar to sunlight in the UV range.

## Conclusion

In future, addressing the question of whether reduced UV-B reflectance in flowers is a negative side effect or an adaptive response can include comparing species that rely on UV to attract pollinators, and those that do not, to determine whether plants downregulate UV in both insect- and non-insect-pollinated flowers. Previous studies have found that UV reflectance in both leaves and fruits are positively correlated [17], however, unlike flowers that often rely on UV-sensitive animals for pollination, many fruits rely on seed dispersal by mammals, which are not able to detect UV-reflectance [30]. Yet another confounding factor that could be addressed in future studies is the role of UV reflectance in plant-herbivore interactions. Similar to the interaction with pollinators, altering leaf pigmentation in response to changes in UV-B stress can have downstream effects on herbivore behavior. Another potential trajectory for future studies is to compare species adapted to different light regimes. Given that some plants grow only in high-UV exposure (e.g. tropical, open-habitat, alpine) while others’ exposure is less predictable, we could predict that the latter would be more adapted to plasticity in their UV reflectance capacities.

Another trajectory for future studies would be to separate the effects of UV-A and UV-B. While due to technical considerations this study filtered out all UV radiation (< 400 nm), separating the two may offer a more nuanced understanding of plant response to high-energy radiation. This is mainly because UV-A radiation is less harmful and may also play an important role in plant development, while at the same time it is the range more likely to be available for insect visual systems [18].

In sum, our results provide a first empirical evidence for the hypothesis that plants increase UV reflectance to mitigate potential damage of UV-B radiation. These results are to be replicated in other species to further establish this hypothesis, as well as to study this phenomenon in the context of plant-herbivore and plant-pollinator interactions.

## Supplementary information

**Additional file 1.**

**Additional file 2: Figure S1.** Spectral Power Distribution: Big Apple Mystic UVB fluorescent tube and Solar Spectrum. Absolute irradiance (μW/cm^2^/nm), at a lamp distance of 10 cm. Solar spectrum from: Bernhard, G., B. Mayer, G. Seckmeyer, and A. Moise (1997), Measurements of spectral solar UV irradiance in tropical Australia, J. Geophys. Res., 102(D7), 8719–8730.

## Data Availability

The datasets used and analysed during the current study available from the corresponding author on reasonable request.

## Abbreviations

C: Celsius
cm: Centimeter
EPA: Environmental Protection Agency
m: Meter
nm: Nanometer
Tbsp: Tablespoon
UV: Ultraviolet
UV-B: The range of wavelengths 280 – 320 nm
μmol: Micromole
μW: Microwatt
