# Supplementary material for: Ultraviolet radiation changes plant color
Source: BMC Plant Biol. 2020 Jun 3;20:253. doi: 10.1186/s12870-020-02471-8 (PMC7268463; doi:10.1186/s12870-020-02471-8)

Spectral Power Distribution: Big Apple Mystic UVB fluorescent tube and Solar Spectrum

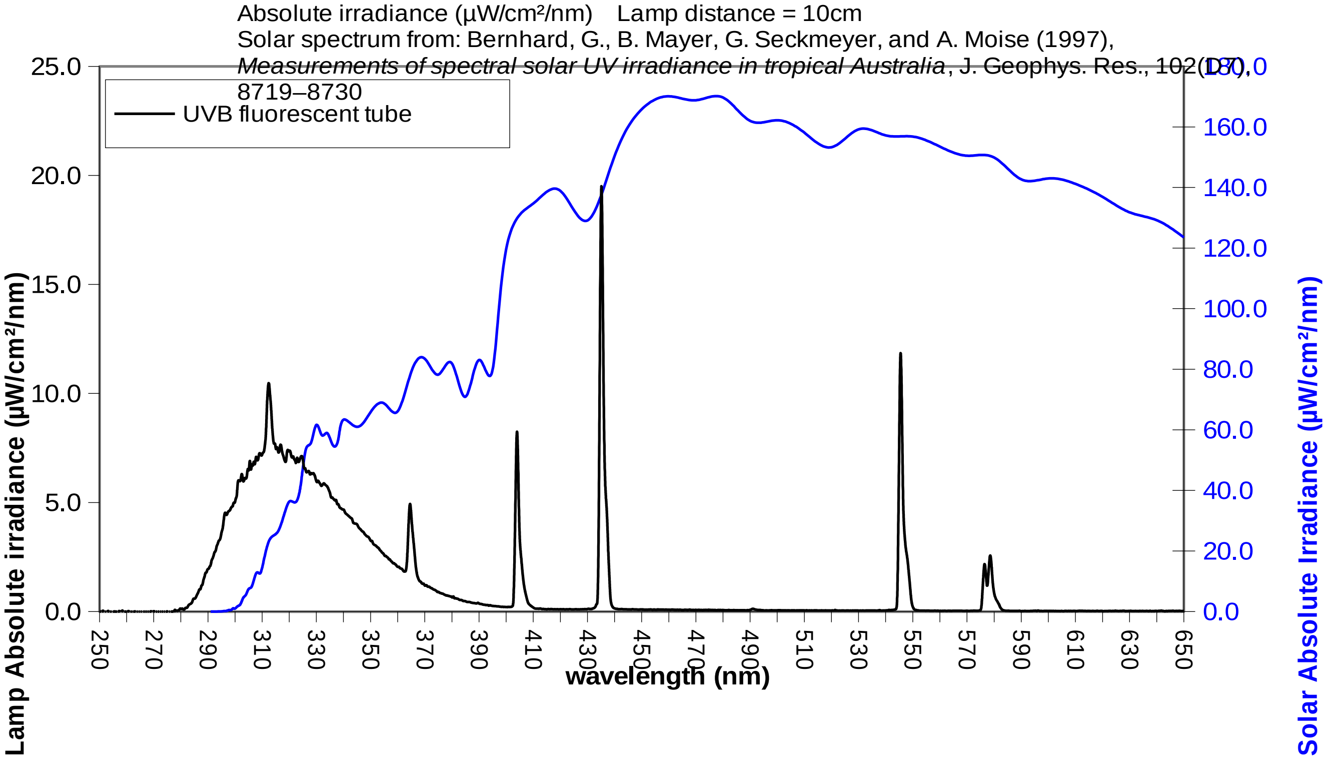

Supplement: Supplementary file 1 [file 12870_2020_2471_MOESM2_ESM.docx]
